# Towards Conformation-Sensitive Inhibition of Gyrase: Implications of Mechanistic Insight for the Identification and Improvement of Inhibitors

**DOI:** 10.3390/molecules26051234

**Published:** 2021-02-25

**Authors:** Dagmar Klostermeier

**Affiliations:** Biophysical Chemistry, Institute for Physical Chemistry, University of Münster, 48149 Münster, Germany; dagmar.klostermeier@uni-muenster.de

**Keywords:** gyrase, mechanism, inhibition, single-molecule FRET, conformational changes

## Abstract

Gyrase is a bacterial type IIA topoisomerase that catalyzes negative supercoiling of DNA. The enzyme is essential in bacteria and is a validated drug target in the treatment of bacterial infections. Inhibition of gyrase activity is achieved by competitive inhibitors that interfere with ATP- or DNA-binding, or by gyrase poisons that stabilize cleavage complexes of gyrase covalently bound to the DNA, leading to double-strand breaks and cell death. Many of the current inhibitors suffer from severe side effects, while others rapidly lose their antibiotic activity due to resistance mutations, generating an unmet medical need for novel, improved gyrase inhibitors. DNA supercoiling by gyrase is associated with a series of nucleotide- and DNA-induced conformational changes, yet the full potential of interfering with these conformational changes as a strategy to identify novel, improved gyrase inhibitors has not been explored so far. This review highlights recent insights into the mechanism of DNA supercoiling by gyrase and illustrates the implications for the identification and development of conformation-sensitive and allosteric inhibitors.

## 1. Introduction: The Bacterial Type IIA Topoisomerase Gyrase

**Figure 1:**
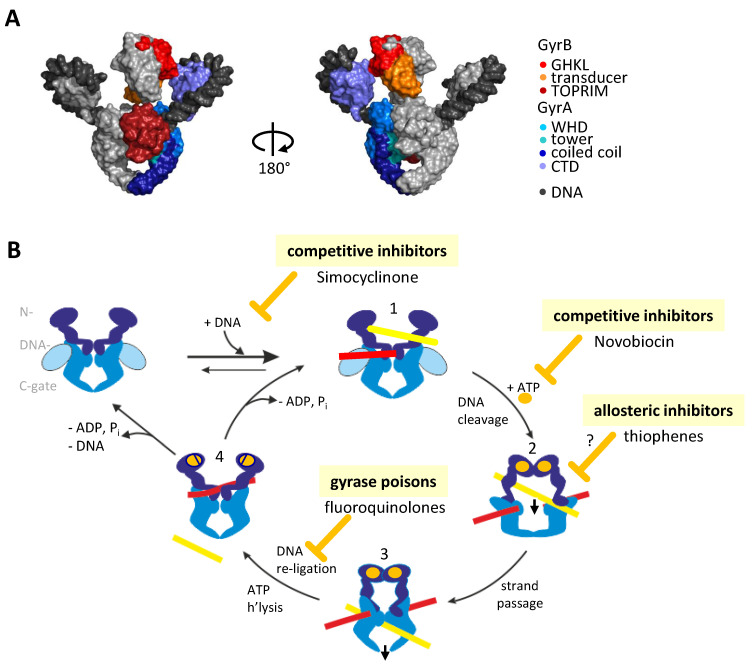
Gyrase function, structure, mechanism, and inhibition. (**A**) Structure of gyrase bound to DNA, the non-hydrolyzable ATP-analog ADPNP, and Ciprofloxacin [16]. One GyrA subunit is colored in blue, one GyrB subunit in red; the second copies are depicted in gray. The DNA (black) binds to the DNA-gate and wraps around both CTDs. GHKL: Gyrase-Hsp90-Histidine Kinase-MutL domain, TOPRIM: topoisomerase–primase domain, WHD: winged-helix domain, CTD: C-terminal domain. Figure from https://www.mdpi.com/1422-0067/19/5/1489, accessed on 3 February 2021. (**B**) Schematic showing the strand-passage mechanism for negative DNA supercoiling by gyrase and steps targeted by inhibitors. Gyrase binds the G-segment (red) at the DNA-gate and a T-segment (yellow) of its DNA substrate (1). Binding of ATP (orange) closes the N-gate and fixes the two segments in a positive node (2). Cleavage of the G-segment at the DNA-gate and passage of the T-segment through the gap (2) converts this positive node into a negative node (3) and decreases the linking number of the DNA by two. The T-segment leaves the enzyme through the C-gate (3), the G-segment is re-ligated, and ATP hydrolysis and product dissociation (4) reset gyrase for the next catalytic cycle. Gyrase activity can be inhibited by competition with ATP binding (e.g., Novobiocin) or DNA binding (e.g., Simocyclinone). Irreversible inhibition is caused by gyrase poisons that stabilize the cleavage complex (e.g., fluoroquinolones). Recently, allosteric inhibition of DNA cleavage by thiophene compounds has been described. The GyrA NTD is shown in blue, the GyrA CTD in light blue. GyrB is depicted in dark blue.

The bacterial type IIA topoisomerase gyrase is the only topoisomerase capable of introducing negative supercoils into DNA in an ATP-dependent reaction [1,2,3,4,5]. In vivo, it is responsible for maintaining a steady-state level of negative supercoils in the genome [6]. In addition, its activity is required during DNA replication and transcription, where it removes positive supercoils ahead of the replication fork or the transcription complex [7,8], respectively (reviewed in [9]). Gyrase is an A_2_B_2_ heterotetramer, formed by the assembly of two GyrA and two GyrB subunits (Figure 1A) [2,10]. GyrA is structured into an N-terminal domain (NTD) and a C-terminal domain (CTD). The NTD consists of a winged-helix domain (WHD), a tower domain, and a coiled-coil domain [11]. The CTD adopts a β-pinwheel fold [12,13]. GyrB contains an N-terminal ATPase domain of the GyrB-Hsp90-histidine/serine protein kinase-MutL (GHKL) type [14], a transducer and a topoisomerase-primase (TOPRIM) domain [15]. The gyrase heterotetramer in its apo state is stabilized by two protein–protein interfaces, termed gates: the DNA-gate is formed by the WHDs of the GyrA subunits and the TOPRIM domains of the GyrB subunit, and the C-gate is composed of the globular domains at the tip of the coiled-coil domains of GyrA [11,16] (Figure 1A). A third gate, the N-gate consisting of the ATPase domains of the GyrB subunits, is open in the apo state of gyrase. The GyrB subunits dimerize on ATP binding [14,16,17], and re-open when both ATP molecules are hydrolyzed [18,19], making the N-gate an ATP-dependent clamp [20,21]. The strand-passage mechanism, postulated in 1979 [22], predicts concerted, large-scale conformational changes during the catalytic cycle of gyrase that couple ATP hydrolysis to DNA supercoiling. Negative supercoiling is initiated by binding of a DNA duplex, termed the G-segment, at the gyrase DNA-gate. Wrapping of the flanking DNA region around the CTD leads to the stabilization of a positive supercoil (Figure 1A,B) [23]. Due to this wrapping, an adjacent region of the same DNA molecule, the T-segment, is placed between the GyrB arms. On ATP binding and N-gate closing, the T-segment becomes trapped in the upper cavity between N- and DNA-gates, above the G-segment. The two catalytic tyrosines located in the WHDs of the GyrA subunits [24] provide the nucleophiles for cleaving both strands of the G-segment. Widening of the gap in the cleaved G-segment by DNA-gate opening is then followed by strand passage, i.e., the translocation of the T-segment through the gap in the G-segment into the bottom cavity between the DNA- and C-gates (Figure 1B). The G-segment is re-ligated, followed by exit of the T-segment through the transiently opening C-gate. ATP hydrolysis and N-gate opening then complete the catalytic cycle. Overall, one strand-passage event leads to the conversion of a positive into a negative supercoil and decreases the number of supercoils (linking number) by two (Figure 1B) [22].

Gyrase is essential for bacteria [25] and absent in humans, which has made it an attractive drug target for the treatment of bacterial infections (reviewed in [26,27,28]). In fact, the genes coding for the gyrase subunits were originally named *nalA* and *cou* as these loci are associated with resistance against the quinolone Nalidixic acid and the coumarin Coumermycin and were renamed only later to *gyrA* and *gyrB* [29]. Inhibition of gyrase is either achieved by competitive inhibitors, or through gyrase poisons that trap cleavage complexes. Unfortunately, many gyrase inhibitors lead to severe side effects, while others rapidly lose their antibiotic activity due to the appearance of resistance mutations. Altogether, there is thus an unmet medical need for novel, improved gyrase inhibitors. Over the last decade, mechanistic studies have revealed the importance of orchestrated conformational changes for DNA supercoiling by gyrase, and have identified key reaction intermediates [18,30,31,32,33,34]. The central role of conformational changes for gyrase activity points to a high potential for allosteric and conformation-sensitive inhibition. Indeed, first inhibitors have recently been shown to bind distant from ATP- and DNA-binding sites and to inhibit gyrase through allosteric effects [35,36]. However, the full potential of interfering with gyrase conformational changes as a strategy to identify novel, improved inhibitors has not been explored so far. This review highlights recent insights into the mechanism of DNA supercoiling by gyrase and illustrates the implications for the identification and development of conformation-sensitive and allosteric inhibitors. These considerations also have ramifications for inhibition of the related bacterial type IIA topoisomerase Topo IV.

## 2. Inhibiting Gyrase: Current Strategies

Inhibition of gyrase activity is achieved through two principal avenues: either by non-covalently binding competitive inhibitors that interfere with ATP or DNA binding, or by gyrase poisons that interact with gyrase in the DNA-bound form and stabilize cleavage complexes (Figure 1B). While competitive inhibition is reversible, the stabilization of cleavage complexes is essentially irreversible, hence such compounds are termed gyrase poisons.

**Figure 2:**
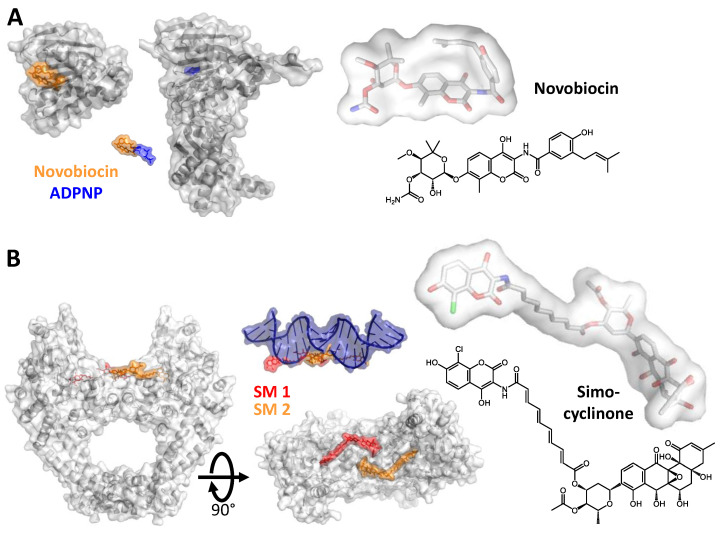
Competitive inhibition. (**A**) Left: Structures of the 24 kDa ATPase fragment of Gyr B with Novobiocin bound [48] and of a 43 kDa fragment bound to ADPNP [49]. Novobiocin (orange) binds to the GHKL domain of the GyrB subunit and acts as a competitive inhibitor by blocking the ATP binding site (occupied by ADPNP, blue). Right: Structure of Novobiocin in stick representation (top) and chemical formula (bottom). PDB-IDs: 4uro (24 kDa fragment of *Staphylococcus aureus* GyrB with Novobiocin), 3zkb (43 kDa fragment of *Mycobacterium tuberculosis* GyrB with ADPNP). (**B**) Left: Structure of the GyrA NTD with two Simocyclinone molecules bound at the DNA-gate of GyrA (red, orange) [44]. The top view (bottom) shows how the compounds block the DNA binding site. The superposition with the DNA (as bound to GyrA in 2xct, top, ref. [50]) shows the overlapping binding sites. SM 1 and SM 2 are the two inhibitor molecules bound to the GyrA dimer. Right: Structure of Simocyclinone in stick representation (top) and chemical formula (bottom). PDB-IDs: 4ckl (*Escherichia coli* GyrA NTD with Simocyclinone), 2xct (*S. aureus* GyrBA fragment comprising the GyrA NTD and the GyrB TOPRIM domain, in complex with DNA and Ciprofloxacin).

An example for a competitive inhibitor that interferes with ATP binding is the aminocoumarin Novobiocin [37,38]. The competitive effect results from an overlap of its binding site in the GHKL domain of GyrB with the nucleotide binding site [2,37,38,39,40,41,42] (Figure 2A). Despite their inhibitory effect on gyrase [1] and their antibiotic activity, the medical use of (amino-)coumarins is limited by their low solubility, low activity against Gram-negative bacteria, and their cytotoxicity, caused by binding to other GHKL-ATPases [27]. Simocyclinones are competitive inhibitors that prevent DNA binding to gyrase by binding to the curved surface at the DNA-gate that serves as a binding site for the G-segment [43,44,45] (Figure 2B). Simocyclinones are bipartite molecules that contain an aminocoumarin and a polyketide part, held apart by a tetraene linker and a sugar moiety. The aminocoumarin and polyketide moieties bind to different GyrA subunits of the GyrA dimer, acting as a non-covalent inter-subunit crosslinker across the DNA-gate [44]. Due to cross-reactions with human topoisomerase II, simocyclinones are cytotoxic for human cells [46,47].

**Figure 3:**
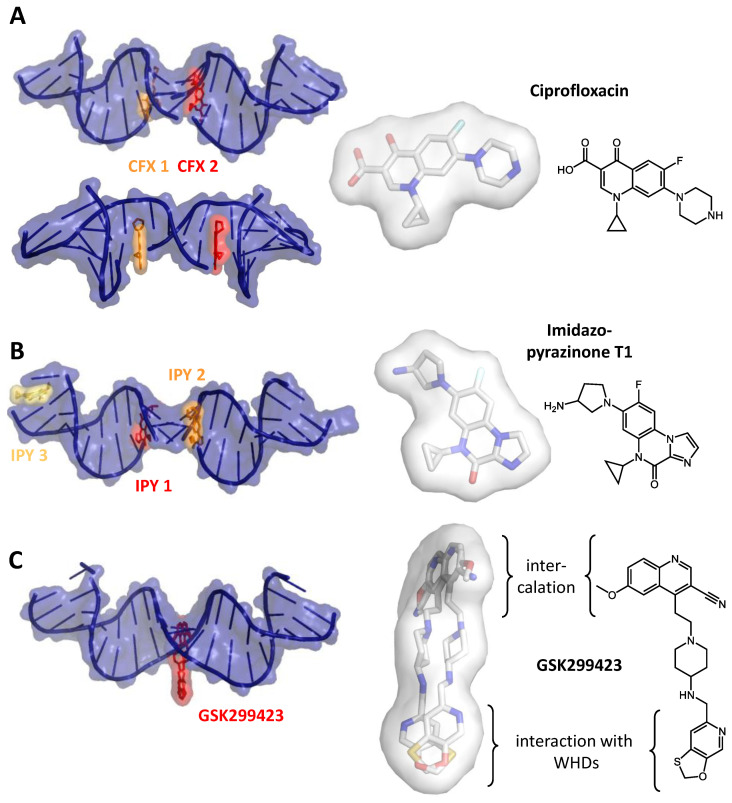
Gyrase poisons. Left: Binding modes. Center, right: Inhibitor structures in stick representation and chemical formula. (**A**) The fluoroquinolone Ciprofloxacin (CFX 1, CFX 2; orange, red) intercalates into the DNA to the left and to the right of the cleavage site [50]. The DNA (blue) is shown in the standard orientation of GyrA (top, see Figure 2B) and rotated (bottom) to highlight the intercalation. (**B**) The Imidazopyrazinone T1 (IPY 1, IPY 2, orange, red) intercalates into the DNA in a similar mode to fluoroquinolones (see panel (**A**)) [57]. A third molecule (IPY 3, yellow) is bound at the end of the DNA. (**C**) The NBTI GSK299423 binds on the two-fold symmetry axis of the GyrA dimer and contacts the DNA-gate of GyrA (not shown) through the oxathiolopyridine moiety, and to the G-segment through the quinoline [50]. Two alternate conformations of the NBTI were modeled. PDB-IDs: 2xct (*S. aureus* GyrBA fragment bound to Ciprofloxacin), 6fqm (*S. aureus* GyrBA fragment with Imidazopyrazinone T1), 2xcs (*S. aureus* GyrBA fragment with GSK299423).

**Figure 4:**
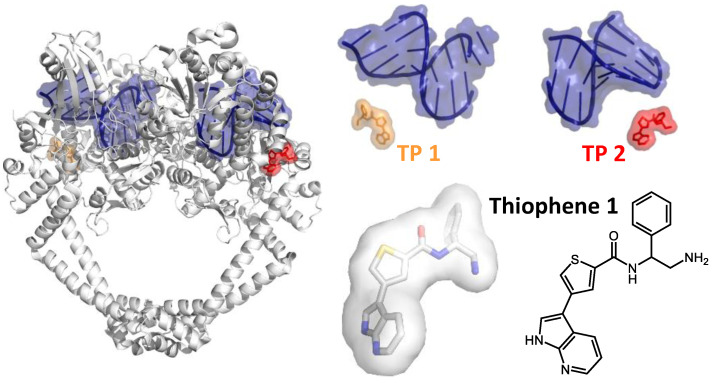
Allosteric inhibition. Left: Structure of a GyrBA fragment bound to DNA and two molecules of Thiophene 1 (orange, red) [35]. Thiophene 1 does not contact the DNA (blue) and inhibits DNA cleavage allosterically. Right: DNA and the position of the two Thiophene 1 molecules (TP 1, TP 2; top) and structure of Thiophene 1 in stick representation and chemical formula. PDB-ID: 5npk (*S. aureus* GyrBA fragment with Thiophene 1).

Gyrase poisons, on the other hand, stall the enzyme on the DNA. Stalled cleavage complexes are detrimental for the cell because they lead to the accumulation of double-strand breaks in the genome and ultimately to cell death [51,52,53]. Structural studies have revealed three distinct binding sites for gyrase poisons [28]. Two of these sites are located near the DNA cleavage site, the third site is remote. The first class of poisons binds directly at the DNA cleavage site (site 1). Due to the two-fold symmetry of gyrase, two of these sites are present, and two inhibitor molecules are bound per heterotetramer. Gyrase poisons intercalate between the nucleobases adjacent to the scissile phosphodiester bonds, which leads to steric interference with the religation reaction. Among the antibiotics targeting site 1 are quinolones, such as Nalidixic acid, and fluoroquinolones, such as Ciprofloxacin (Figure 3A) [50]. Due to the wide-spread appearance of resistance mutations, clustered in quinolone-resistance-determining regions [54,55], and severe, possibly permanent side effects in patients, the clinical use of fluoroquinolones in Europe has been restricted recently (https://www.ema.europa.eu/en/medicines/human/referrals/quinolone-fluoroquinolone-containing-medicinal-products (accessed on 3 February 2021)). Examples for other compounds binding to site 1 include the anti-cancer drug Etoposide [56] and inhibitors of the imidazopyrazinone family (Figure 3B) [57]. So-called “novel bacterial topoisomerase inhibitors” (NBTIs) also inhibit DNA cleavage [50,58], but their binding site (site 2) is located on the two-fold symmetry axis of gyrase [50], such that one inhibitor is bound to the gyrase heterotetramer. NBTIs are chemically diverse but share a bipartite architecture that enables them to interact simultaneously with GyrA and the DNA bound. The NBTI GSK299423, identified by Glaxo–Smith–Kline through screening of a compound library for gyrase inhibitors, consists of a quinoline-nitrile moiety fused to an oxathiolo-pyridine [50]. Its quinoline-nitrile group intercalates between the central base pairs of the DNA bound at the DNA-gate (i.e., not at cleavage site), and the oxathiolo-pyridine part is inserted into a pocket that is transiently formed between the WHDs of GyrA [50] (Figure 3C). Effectively, NBTIs thus non-covalently crosslink GyrA with the G-segment. In addition to their effect on DNA cleavage, they inhibit the DNA-dependent ATPase activity through an allosteric mechanism [36]. Due to the two-fold symmetry of the gyrase heterotetramer, NBTIs can bind to site 2 in two possible orientations (Figure 3C). Consequently, their electron density is averaged in many crystal structures, which complicates structure-based drug design. Only recently, a structure of an NBTI bound to gyrase was reported that showed the inhibitor in a single orientation [59]. In addition to its potential use for drug design, this structure rationalizes how NBTIs induce single-strand breaks, in contrast to the double-strand breaks induced by site 1 binders. Although the binding site of NBTIs is close to site 1 occupied by fluoroquinolones, they are active against fluoroquinolone-resistant bacterial strains, including methicillin-resistant *Staphylococcus aureus* (MRSA) [58,60,61]. The interaction site between the GyrA WHDs is not conserved in human topoisomerase II, and NBTIs are therefore specific for gyrase [50]. A third class of poisons is represented by the recently discovered thiophenes that stabilize cleavage complexes with either single- or double-strand breaks. These compounds bind remotely from the ATPase and DNA-binding sites (Figure 4) [35] (site 3). They interact with a groove on the surface of gyrase, located between the TOPRIM and WH domains of GyrB and GyrA, respectively. Residues forming this binding site, termed the “hinge pocket” [35], are conserved among gyrases, with the exception of the *M. tuberculosis* enzyme. The binding site is absent in the apo enzyme, implying that thiophenes bind to a catalytic intermediate in which the binding site is transiently formed. It has been suggested that they inhibit gyrase allosterically by preventing conformational rearrangements at the DNA-gate (Figure 4) [35]. The increasing knowledge on conformational changes of gyrase during DNA supercoiling and on the conformation of catalytic intermediates provides a yet-to-be-explored basis for the identification and design of allosteric inhibitors targeting distinct steps in the supercoiling cycle.

## 3. DNA Supercoiling Requires a Series of ATP- and DNA-Induced Conformational Changes of Gyrase

**Figure 5:**
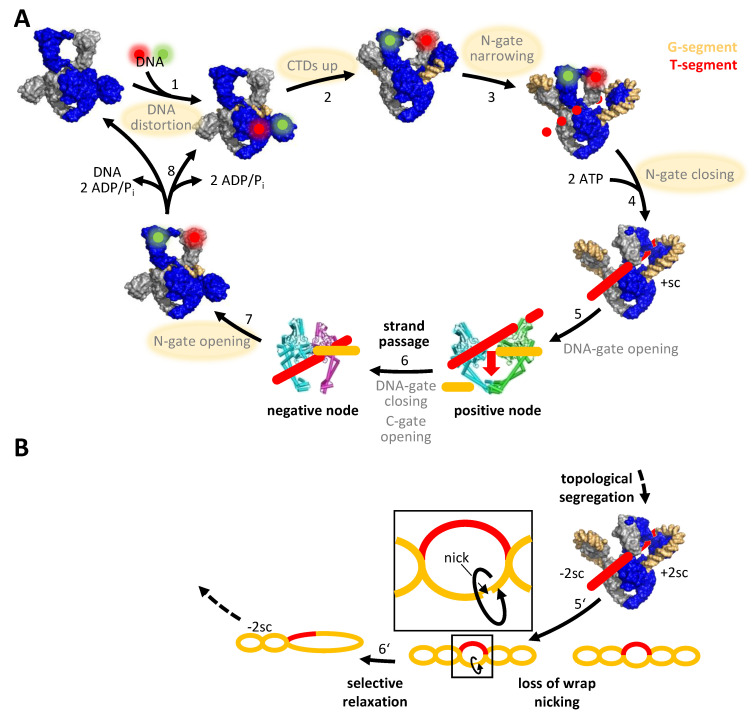
Conformational changes in the catalytic cycle of gyrase. (**A**) Conformational changes during DNA supercoiling by gyrase, as delineated by single-molecule FRET. Green, red: donor- and acceptor fluorophores for FRET experiments. One GyrA and GyrB subunit is shown in blue, the second in gray. Green and red spheres denote the position of donor and acceptor fluorophores to probe conformational changes of the DNA (1), the position of the CTD (2), or the conformation of the N-gate (3, 4, 7). Conformational changes detected by FRET are highlighted in yellow. The other conformational changes indicated, i.e., opening and closing of the DNA- and C-gates, have been postulated but were not yet observed by FRET. Crystal structures of GyrA in which one of the gates is open illustrate that opening of these gates is possible in principle. According to the strand-passage model, strand passage (6) converts a positive node into a negative node, decreasing the linking number of the DNA by two. Figure from https://www.mdpi.com/1422-0067/19/5/1489, accessed on 3 February 2021. (**B**) Gyrase with a single catalytic tyrosine catalyzes DNA supercoiling in the absence of strand passage in steps of two. This enzyme undergoes the same cascade of DNA- and nucleotide-induced conformational changes as does wild-type gyrase, i.e., DNA distortion, DNA-dependent movement of the CTD, narrowing of the N-gate, and ATP-dependent closing of the N-gate (steps 1–4 in panel (**A**)). DNA supercoiling in the absence of double-strand cleavage and strand passage can be rationalized by the capture of two positive supercoils, followed by topological segregation and selective relaxation of these supercoils (steps 5 and 6). Selective relaxation is brought about by nicking and rotation of the DNA around the phosphodiester bonds in the contiguous, non-cleaved strand (inset). Afterwards, ATP hydrolysis, N-gate opening and product release (steps 7, 8 in panel (**A**)) reset gyrase for further catalytic cycles. Only the steps deviating from the strand-passage mechanism shown in panel (**A**) are depicted. As the structural basis for the capture of two supercoils by gyrase is unknown, the schematic shows only the DNA sketch. +sc: positive supercoil, +2 sc: two positive supercoils, −2 sc: two negative supercoils, CTD: C-terminal domain.

The detailed knowledge on enzymatic mechanisms provides insight into new avenues for inhibitor design. X-ray crystallography and cryo-electron microscopy have provided ample structural information on gyrase over the last 30 years. Structures available range from subunit fragments, such as the GyrB ATPase domain [14], the TOPRIM domain [15], the GyrA NTD [11], or the GyrA CTDs [13] to full-length gyrase in complex with the non-hydrolyzable ATP-analog ADPNP, double-stranded DNA and a fluoroquinolone (Ciprofloxacin) or NBTI (Gepotidacin) inhibitor bound [16,17]. None of the few high-resolution structures of possible reaction intermediates comprises the entire enzyme without a bound inhibitor, though, although such structures would be of great value for inhibitor development. Single-molecule Förster resonance energy transfer (FRET) has been instrumental in dissecting conformational changes of gyrase during the supercoiling reaction (reviewed in [62]) and has substantially refined the original understanding of the strand-passage mechanism (reviewed in [30]). These studies delineated the nucleotide-driven conformational changes of the N-gate inferred from biochemical data and revealed that the DNA substrate plays a hitherto unappreciated active role in driving gyrase conformational changes. They also identified novel reaction intermediates. Single-molecule FRET has revealed that the G-segment becomes distorted on binding to the DNA-gate [34] (Figure 5A). The DNA regions emanating from the DNA-gate contact the CTDs, which induces their upward movement [33]. Wrapping of the DNA around the entire CTD perimeter then triggers narrowing of the N-gate [18,32]. Gyrase with a narrowed N-gate is a novel intermediate, which may be relevant for intramolecular strand passage and thus gyrase-specific [18]. The narrowed N-gate closes on ATP binding [18,63]. At this point, the T-segment is bound in a fixed orientation above the G-segment [16]. According to the strand-passage mechanism, G-segment cleavage and DNA-gate opening would now allow for passage of the T-segment through the gap in the G-segment, followed by G-segment religation and exit of the T-segment through the C-gate. Different conformations observed in the crystal structure of *Bacillus subtilis* GyrA suggest that the DNA- and C-gates should, in principle, be able to open [31]. Structures of other type IIA topoisomerases have indeed captured the DNA- or C-gate in open states [64,65,66]. However, opening of the DNA- and C-gates of gyrase have never been observed during the supercoiling reaction in real time. Single-molecule FRET demonstrated that hydrolysis of both ATP molecules enables N-gate re-opening, and product release then resets gyrase for subsequent catalytic cycles [18,19].

**Figure 6:**
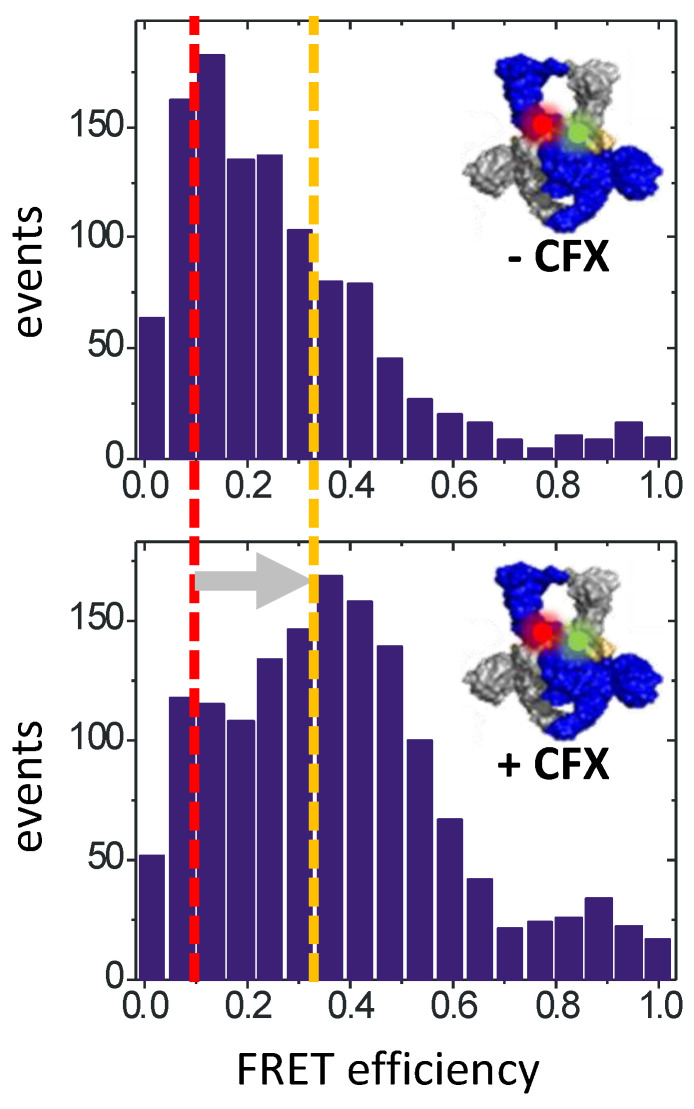
Single-molecule FRET unravels inhibitory mechanisms. Single-molecule FRET histograms of gyrase-bound DNA carrying donor and acceptor fluorophores on opposite sides of the cleavage site. DNA bound to gyrase exists in two conformations, characterized by FRET efficiencies of 0.13 (red line) and 0.3 (orange line; top). The low-FRET state corresponds to DNA that is severely distorted from B-form geometry. Ciprofloxacin (CFX) binding shifts the distribution to the high-FRET species (gray arrow; bottom), indicating that CFX counteracts DNA distortion by gyrase. Such FRET assays can be adapted to screen for inhibitors of conformational changes. Figure modified after [34].

Overall, single-molecule FRET has provided detailed insights into the molecular motions of gyrase during supercoiling and has helped define the relevant conformational states. The potential of FRET as a technique and of the insight gained from FRET studies for gyrase inhibition is manifold. Firstly, each of the conformational states identified constitutes a possible target for conformation-sensitive and/or allosteric inhibitors. Second, only minor modifications in labeling strategies are needed to use the donor/acceptor-labeled constructs established for single-molecule studies as a tool for high-throughput screens of compound libraries to identify inhibitors blocking individual conformational changes. Thirdly, FRET experiments, both on the single-molecule and the ensemble levels, can help understand inhibitory mechanisms of small molecules. This is exemplified by single-molecule FRET experiments on the gyrase poison Ciprofloxacin, which have shown that this compound interferes with the distortion of the G-segment at the DNA-gate [34] (Figure 6). Despite its enormous potential, (single-molecule) FRET has not been fully exploited yet to identify gyrase inhibitors that interfere with conformational changes, or to dissect the mechanism of action of inhibitors identified by other means.

## 4. The Role of Symmetry: An Alternative Mechanism for DNA Supercoiling without Strand Passage

The gyrase heterotetramer shows two-fold symmetry, and contains two CTDs, two ATPase domains, and two catalytic tyrosines. Interestingly, gyrase can catalyze DNA supercoiling with just a single CTD, which is sufficient to stabilize a positive supercoil [67,68]. The role of the second CTD is unclear. The presence of two CTDs may increase the probability to capture the positive supercoil by providing two possibilities to wrap DNA around one of the two CTDs, either left or right of the G-segment [67]. Alternatively, the presence of a second CTD may just be a consequence of the symmetry of the enzyme. Gyrase also catalyzes DNA supercoiling when only one of the ATPase domains in the GyrB subunits is functional [19]. In such a variant, supercoiling is coupled to binding and hydrolysis of a single ATP: ATP binding to the functional site triggers N-gate closing, and its hydrolysis immediately leads to re-opening of the N-gate [19]. Binding of the second ATP may increase N-gate stability and may act as a timer to ensure efficient coupling of the nucleotide cycle to DNA supercoiling.

According to the strand-passage mechanism, the two catalytic tyrosines are both required for double-strand cleavage. However, we recently showed that a variant lacking one of these tyrosines only cleaves one of the two strands in the G-segment, but still catalyzes DNA supercoiling in steps of two, although the reaction occurs more slowly compared with wildtype gyrase [68]. Interestingly, this variant undergoes the same series of conformational changes in the beginning of the supercoiling cycle as does the wildtype [68], although its interactions with the DNA must be different. Supercoiling by gyrase with a single catalytic tyrosine must occur through a nicking-closing mechanism [2,68,69,70], without strand passage. We have proposed a model in which gyrase captures two positive supercoils in the DNA, followed by their selective relaxation (Figure 5B) [68,71]. It is currently unclear if this mechanism is a back-up mechanism that only occurs when one of the two tyrosines is missing, if gyrase uses either mechanism, or if negative supercoiling generally occurs without strand passage. In any case, the capability of gyrase to supercoil DNA with just a single cleavage event suggests that only inhibitors interfering with cleavage in both GyrA subunits will abrogate gyrase activity completely. 

While DNA supercoiling by gyrase is possible without strand passage, ATP-dependent relaxation and decatenation, the hallmark reactions of the related enzymes eukaryotic topoisomerase II and bacterial topoisomerase IV, require two catalytic tyrosines and strand passage [68]. The mechanistic differences between these structurally similar enzymes might provide hitherto unappreciated strategies to inhibit gyrase (and/or Topo IV) without affecting human topoisomerase II.

## 5. Species-Specific Elements Modulate Gyrase Activity

**Figure 7:**
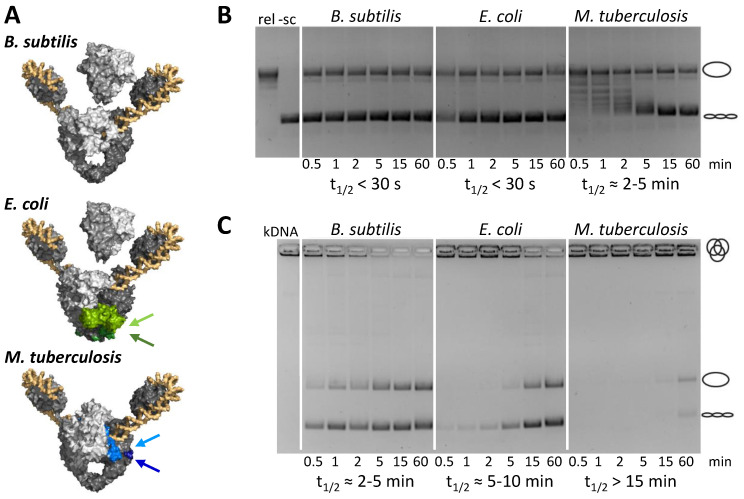
Species-specific elements in gyrase modulate enzymatic activities. (**A**) *E. coli* and *M. tuberculosis* gyrase contain insertion elements in GyrA and GyrB (depicted in light and dark green for *E. coli*, light and dark blue for *M. tuberculosis*, see arrows) that are absent in *B. subtilis* gyrase (top). These elements mediate additional interactions between the subunits. *E. coli* gyrase is shown in complex with ADPNP and the NBTI Gepotidacin, as observed by cryo-EM (PDB-ID 6rkw). The structure for *B. subtilis* gyrase is a homology model (see ref. [68] for details). The model of full-length *M. tuberculosis* gyrase represents the crystal structure of *M. tuberculosis* gyrase lacking the CTDs (PDB-ID 6gau), with the CTDs placed according to their position in the cryo-EM structure of *E. coli* gyrase. (**B**) The species-specific insertion elements modulate the supercoiling activity: *B. subtilis* and *E. coli* gyrase are efficient supercoiling enzymes that supercoil half of the DNA in less than 30 s. In contrast, *M. tuberculosis* gyrase catalyzes supercoiling more slowly, with a half-life of 2–5 min. The supercoiling endpoints also differ [74]. (**C**) The insertion elements also modulate the decatenation activity of the different gyrases. *B. subtilis* gyrase decatenates kinetoplastid DNA (kDNA) fastest with a half-life of 2–5 min; the half-life for *E. coli* gyrase is 5–10 min. *M. tuberculosis* gyrase is also the slowest enzyme in decatenation, with a half-life of >15 min. Figure modified from [74].

Although gyrases share the common type IIA topoisomerase scaffold, enzymes from different bacteria contain species-specific elements [72,73,74]. A comparison of gyrases from *Escherichia coli*, *B. subtilis*, and *M. tuberculosis* reveals that the *B. subtilis* enzyme is a minimal version. Compared to *B. subtilis* gyrase, the *E. coli* enzyme, commonly viewed as the archetype of a gyrase, contains a 170-amino acid insertion in the TOPRIM domain of GyrB [72] and a 34-amino acid insertion of hitherto uncharacterized function in the coiled-coil domain of GyrA. *M. tuberculosis* gyrase contains a 4-amino acid insertion in the tower domain of GyrA and a short insertion in the GHKL domain of the GyrB subunit [73]. In both enzymes, the insertions in the two subunits interact in the gyrase heterotetramer [72,73] (Figure 7A). More importantly, they modulate the supercoiling and decatenation activities of these enzymes (Figure 7B,C). Gyrase activities are further modulated by sequence and structural variations in the GyrA CTDs [13,75,76,77,78]. As a result, enzymes from different bacteria have different supercoiling set-points, and a different balance between wrapping-dependent supercoiling and wrapping-independent decatenation of DNA [74,75]. This species-specific modulation of gyrase activity and the potential variations in the underlying mechanisms have important ramifications for inhibitor identification and structure-guided drug design: on one hand, these differences can be exploited to identify compounds that target the species-specific insertion, offering the possibility to develop highly effective gyrase inhibitors specific for a particular enzyme. On the other hand, the variations between species will complicate or even make it impossible to transfer inhibitory strategies from one enzyme to another, impeding the identification of a pan-inhibitor from studies with just a single model enzyme.

## 6. Implications for Gyrase Inhibition: Conclusions and Outlook

Gyrase numbers per *E. coli* cell have been estimated to approximately 600 [79], corresponding to average in vivo concentrations of the order of 10 nM. Cellular ATP concentrations are in the low millimolar range [80], and the DNA concentration has been estimated to 11–18 mg/mL [81,82], putting potential gyrase binding sites also into the millimolar concentration range [83]. Thus, competitive gyrase inhibitors targeting the ATP- or DNA-binding sites are needed in high concentrations to reach their inhibitory effect. Furthermore, they are prone to off-target effects by cross-reacting with other ATP-binding or DNA-binding proteins. On the other hand, the stabilization of cleavage complexes by gyrase poisons, although powerful due to its bactericidal effect, is associated with side effects due to the ability of these compounds to intercalate into DNA. Thiophenes with their allosteric mode of inhibition open up a new avenue for the identification of gyrase inhibitors. Allosteric inhibitors interfering with ATP binding and hydrolysis or DNA binding and cleavage are inherently more powerful than competitive inhibitors. To inactivate all gyrase molecules, high-affinity binders would only be required in nanomolar concentrations in the cell. High-specificity binders would generate fewer off-target effects. Furthermore, the problems associated with loss of antibiotic activity due to resistance may be reduced: hinge regions are essential for gyrase activity and might be under selective pressure. Thus, lower mutation frequencies can be expected in these regions. 

**Figure 8:**
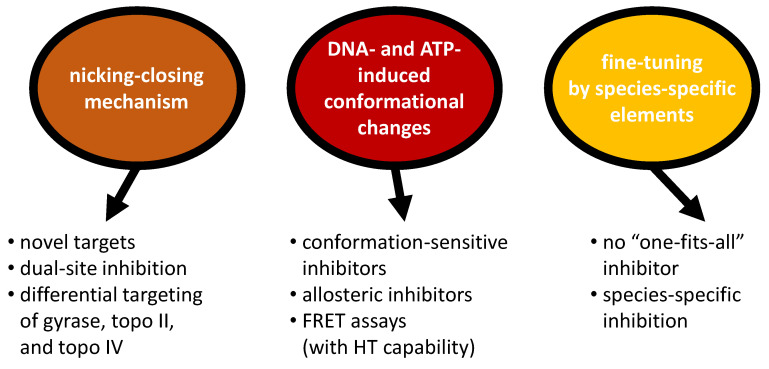
Novel strategies for gyrase inhibition and inhibitor identification. Insight into the mechanism of DNA supercoiling by gyrase opens up novel pathways for gyrase inhibition. See “Implications for gyrase inhibition: Conclusions and Outlook” for details and discussion. HT: high throughput, topo II/IV: topoisomerase II/IV.

Structural insight into reaction intermediates during DNA supercoiling and into gyrase conformational dynamics provides valuable information on potential avenues for gyrase inhibition (summarized in Figure 8). Single-molecule FRET has identified a cascade of concerted conformational changes during DNA supercoiling and has helped to define the global conformations of the relevant catalytic intermediates. Each of these conformations contains unique binding sites for small molecules, and thus is a potential drug target. Knowledge on these conformational states and on the events that trigger their inter-conversion thus provides a rich basis for the development of conformation-sensitive and allosteric inhibitors. The identification of hinge regions as promising binding sites for such inhibitors would undoubtedly benefit from high-resolution structural information on such conformational intermediates. Recent developments in cryo-EM hold great promise for advancing structure-guided drug design, particularly on large, flexible enzymes such as gyrase. The structure of full-length *E. coli* gyrase in complex with DNA, ADPNP, and Gepotidacin [17] serves as a first example that cryo-EM can be employed for the analysis of small-molecule inhibitor complexes of gyrase. While structural studies will not be able to provide information in higher-throughput formats in the near future, the experimental strategies used to monitor conformational changes on the single-molecule level can readily be adapted for FRET ensemble assays with high-throughput capabilities. Such assays enable the screening of large compound libraries for inhibition of individual gyrase conformational changes. Structural approaches and dynamic FRET studies thus complement each other in unravelling the molecular basis for inhibitory effects the inhibitory mechanism. 

The observation that gyrase can supercoil DNA in the presence of just a single catalytic tyrosine, a single functional ATPase site or a single CTD, also has important consequences for gyrase inhibition. Complete inhibition of enzymatic activity and a strong antibiotic effect may require dual-site inhibition, i.e., binding of inhibitors to both sides of the enzyme. Furthermore, the fact that ATP-dependent decatenation and relaxation require both catalytic tyrosines and rely on double-strand cleavage and strand passage might simplify selective inhibition of gyrase without affecting topoisomerase II (or topoisomerase IV, although this enzyme is often targeted simultaneously with dual inhibitors to reduce the probability of resistance [84]).

Finally, the observation that species-specific elements modify the enzymatic properties of gyrase suggests that it might be difficult to identify a “one-fits-all” pan-inhibitor that efficiently inhibits gyrases from a wide range of bacterial species. At the same time, purposefully targeting the species-specific elements opens up the possibility for highly efficient and selective inhibition a particular gyrase. A currently employed experimental platform for structure-guided drug design by X-ray crystallography is based on the *S. aureus* gyrase core, a truncated fusion protein in which the GyrA NTD and the GyrB TOPRIM domains are covalently linked into a single polypeptide chain [50] (reviewed in [28]). It should be noted that this enzyme is not able to catalyze DNA supercoiling and may not be able to access the functionally relevant conformational intermediates in the catalytic cycle. While such tailored constructs are suitable to investigate the structural basis of known gyrase inhibitors and for structure-based drug design, identifying novel conformation-selective inhibitors requires structural models of the complete enzyme, e.g., from cryo-EM. More importantly, lead compounds targeting *S. aureus* gyrase may be difficult to adapt for gyrases from other bacteria. Both compound screening and structure-based drug design would undoubtedly benefit from a broader basis early on in the drug discovery process by employing gyrases from several bacteria in parallel in the screening cascade. Candidates obtained with only a subset of these enzymes might hold potential for the development as high-efficiency, high-specificity inhibitors, whereas candidates obtained with different gyrases could be considered for further development to a pan-inhibitor.
